# Detection of New Delhi metallo-β-lactamase (*bla*_NDM_) and oxacillinase (*bla*_OXA-48_) genes among carbapenem-resistant Enterobacteriaceae (CRE) in Jazan Region, Saudi Arabia

**DOI:** 10.1038/s41598-026-49160-4

**Published:** 2026-04-24

**Authors:** Soheir A. A. Hagras, Gharieb S. El-Sayyad, Marwa Yousry A. Mohamed, Hani A. Ozbak, Mahitab M. Wahby, Sara Ahmed Eltigani, Neamat Hanem M. I. Dorra, Samar Elshahidy, Mostafa A. Abdel-Maksoud, Salman Alrokayan, Hassan A. Rudayni, Yasser Q. Majrabi

**Affiliations:** 1 Pharmacy Department, Alnahda College, Al Munsiyah, Riyadh, 13255 Kingdom of Saudi Arabia; 2 Department of Biology, College of Science, Imam Mohammad Ibn Saud Islamic University (IMSIU), Riyadh, 11623 Saudi Arabia; 3 Department of Clinical Laboratory Sciences, College of Applied Medical Sciences, Taibah University, P.O. Box 344, Al-Madinah Al-Monawra, 41411 Kingdom of Saudi Arabia; 4 Faculty of Medicine, Misr University for Science and Technology (MUST), 6th of October, Giza, 3237101 Egypt; 5 Department of Laboratory, Jazan General Hospital, Jazan, Kingdom of Saudi Arabia; 6 Microbiology and Immunology Department, Faculty of Pharmacy, Sinai University Kantara Branch, Ismailia, Egypt; 7 Dr. Sulaiman Al-Habib Medical Group, Riyadh, Kingdom of Saudi Arabia; 8 Faculty of Science, Suez Canal University, Ismailia, Egypt; 9 Research Chair of Biomedical Applications of Nanomaterials, Biochemistry Department, College of Science, King Saud University, Riyadh, Kingdom of Saudi Arabia; 10 Ministry of Health, Jazan, Kingdom of Saudi Arabia

**Keywords:** Carbapenem-resistant Enterobacteriaceae (CRE), *Bla*_NDM_, *Bla*_OXA-48_, *K. pneumoniae*, Jazan, Saudi Arabia

## Abstract

Carbapenem-resistant Enterobacteriaceae (CRE) are critical priority pathogens due to their resistance to carbapenems, the last-resort antibiotics, and their rapid spread in healthcare facilities. The study investigates the epidemiological and molecular characteristics of CRE isolates in the Jazan region, Saudi Arabia. Clinical specimens were collected from patients admitted to Jazan General Hospital between December 2023 and May 2024. Identification of the isolates was done by the VITEK 2 system, and their susceptibility to antibiotics was determined on the MicroScan WalkAway platform according to the EUCAST guidelines. The E-test was used to confirm the CRE isolates. Molecular detection of carbapenemase genes (*bla*_IMP_, *bla*_VIM_, *bla*_NDM_, *bla*_KPC_ and *bla*_OXA-48_) was conducted using the GeneXpert Carba-R assay using the GeneXpert®. A total of 426 Enterobacterial clinical isolates were collected from patients; among them, 53 (12.4%) were phenotypically found to be CRE; among such isolates, 14 (26.4%) were found to be carbapenemase-positive Enterobacteriaceae (CPE), and 39 (73.6%) were carbapenemase-negative Enterobacteriaceae (CNE). Among the CPE strains, *Klebsiella pneumoniae* was the predominant organism (42.9%), followed by *Escherichia coli* (28.6%), *Enterobacter cloacae* (21.4%), and *Serratia marcescens* (7.1%). Molecular analysis revealed a high prevalence of the *bla*_NDM_ gene (71.4%), with *bla*_OXA-48_ detected in 42.9% of isolates. The *bla*_VIM_ was identified in only 7.1%, while neither the *bla*_KPC_ nor *bla*_IMP_ is detected. For the CNE strains, *K. pneumoniae* was most common (62%), of which 15% produced extended-spectrum β-lactamase (ESBL). The isolates were all highly resistant to penicillin and β-lactam/β-lactamase inhibitors, moderately susceptible to fluoroquinolone (33%) and aminoglycosides (49%), and most susceptible to tigecycline (79%). The *bla*_NDM_ was mainly found among ICU patients, while *bla*_OXA-48_ was most frequently identified among pediatric patients. The ICU admission with invasive devices, and prior broad-spectrum antibiotic exposure emerged as the main risk factors for the evolution of CPE. Multiple comorbidities; particularly malignancy, diabetes, and surgery; further increased vulnerability to CPE infections. Jazan city has a lower prevalence of CPE, while non-carbapenemase resistance is most predominant, reflecting regional epidemiological heterogeneity, which is important for regional stewardship and control.

## Introduction

Antimicrobial resistance (AMR) is a major global health threat in both developing and developed countries, associated with massively increasing morbidity and mortality rates, in addition to the negative impact on the economy. Such a silent pandemic directly results in 1.3 million annual deaths^1^, and contributed to a total of 4.95 million deaths in 2019^2^. Carbapenems are considered the last resort of antibiotics for the treatment of infections caused by resistant strains and are commonly reserved for severe, life-threatening infections^3–5^. Unfortunately, resistance to such crucial drugs has emerged and is increasing disastrously, especially within the family Enterobacteriaceae.

Carbapenem-resistant Enterobacteriaceae (CRE) easily spreads through contact with infected or colonized people, particularly contact with wounds, contaminated healthcare professionals, or contaminated devices and surfaces^6^. CRE were first reported in the 1990s; however, outbreaks of CRE have been massively increasing and have become problematic in clinical practice, with a death rate exceeding 50%^7–9^. The common treatments for CRE infections commonly include combinations of tigecycline, colistin, and aminoglycosides, which are associated with serious side effects, e.g., ototoxicity and nephrotoxicity, in addition to the emergence of pandrug-resistant strains (PDR)^10^.

Major risk factors for the acquisition of CRE include prolonged hospitalization, previous carbapenem treatment, the use of invasive devices, and underlying comorbidities, in addition to the use of antacids^5,11–13^. A multicenter study reported overall incidence rates of CRE infection to be 4.0, 2.93, and 1.3 per 10,000 discharges in China, the United States, and European countries, respectively^14,15^. Reports from Africa documented an increasing burden of CRE across Africa with evident regional variation, but the limited diagnostic capacity and weak surveillance systems continue to hinder timely detection and access to effective therapy^16^. Akinyemi and Abdelmegid emphasized in their commentary on the value of molecular epidemiology in understanding the transmission of CRE within a One Health framework^17^.

Additionally, Alhaji et al. (2025), reported the circulation of of carbapenemase through human, animal, and environmental reservoirs^18^. The Centers for Disease Control and Prevention (CDC) classified CRE among the five top “urgent hazard level” threats to human health and multidrug-resistant (MDR) infections^19^. Autochthonous CRE infections are very common in the Arabian countries^8,20^. Saudi Arabia specifically is a common destination for millions of expatriates for the purpose of pilgrimage, work, or tourism. Such population characteristics make the Kingdom highly vulnerable to continuous and increasing rates of the dissemination of different infections, including CRE^8^. Alraddadi et al.^21^ conducted a multicenter cohort study for more than 2 years to estimate the CRE prevalence in Saudi Arabia, where they reported that infections caused by CRE are commonly associated with poor outcomes due to treatment failure^21^. Multiple resistance mechanisms have been reported in CRE; the common mechanism is the production of carbapenemases, which inactivate carbapenems and other β-lactam antibiotics, including penicillins and cephalosporins. Carbapenemase genes are carried on plasmids that are easily transmitted from one bacterium to another, provoking the spread of resistance^15^. Three carbapenemase classes are reported based on their amino acid sequence according to the Ambler system; Class A includes KPC and GES for *K. pneumoniae* carbapenemase and Guiana extended-spectrum β-lactamase, respectively. Class B includes the metallo-β-lactamases: New Delhi metallo-β-lactamase (NDM), Imipenemase (imipenem-hydrolyzing) (IMP), and Verona integron-encoded metallo-β-lactamase (VIM). Finally, Class D is the oxacillinase (OXA-48)^9,22,23^.

Enterobacteria may also develop resistance to carbapenems due to chromosomal mutations in a porin gene that limit the ability of carbapenems to reach their target sites, combined with the acquisition or upregulation of a β-lactamase. CRE without carbapenemases are called non-carbapenemase-producing CRE^11,24–26^. Research reported significant variability and diversity in the distribution of resistance mechanisms. The rates and mechanisms among CRE differ from time to time and from area to another^8^, even from unit to another and from patient to another in the same hospital^7,27^.

Accordingly, public health demands addressing CRE to guide management strategies, so continuous monitoring of such pathogens in clinical settings is crucial^12,20,27,28^. Patients can be better managed by being aware of the common mechanisms of resistance and by choosing the best antibiotic therapies. This study aims to generate deep insights into the epidemiology, risk factors, microbiological, and molecular characteristics of CRE in Saudi Arabia.

## Materials and methods

### Ethical approval

This study was conducted at the microbiology lab in Jazan General Hospital, Saudi Arabia. The research protocol was approved by the Research Ethics Committee at Jazan Hospital (No. 23109) on 04/12/2023. The panels approved the research under an expedited review, where no more than minimal risk to study subjects according to 45 CFR 46.110^29^. Patients’ privacy and confidentiality were assured in compliance with the Declaration of Helsinki^30^. Patients’ data were anonymously collected.

### Inclusion criteria

All patients admitted to Jazan General Hospital and hospitalized for more than 48 h were included in this study. A total number of 1150 clinical samples (blood, urine, sputum, stool, and wound specimens) were collected from admitted patients based on the underlying disease. The study extended over 6 months, from December 2023 to May 2024. The following variables were used to identify risk factors contributing to the emergence of CRE: sex, age, underlying diseases, comorbidities, sample type, antibiotic therapy, ICU, and any medical interventions.

### Isolation and identification of CRE strains

Specimens were cultured on MacConkey agar (Oxoid, England). Isolates were examined macroscopically for their cultural characteristics, Gram-stained, and examined microscopically. Biochemical characteristics of bacterial isolates were tested using the Vitek 2 Compact automated system (Biomerieux, France) according to the manufacturer’s guidelines. Briefly; The test organism was suspended in sterile 0.45–0.50% saline to a 0.5 McFarland turbidity and used to inoculate the identification cards, and incubated at 35.5 ± 1.0 °C. Optical readings taken every 15 min, the resulting biochemical reaction pattern was then compared with the system’s database to generate a probability-based organism identification^31,32^.

### Antibiotic susceptibility testing (AST) of the isolates

Antibiotic susceptibility testing (AST) for determining the MIC was performed using MicroScan WalkAway Microbiology ID/AST System (Beckman Coulter, California, USA) according to the manufacturer’s guidelines. Gram‑Negative MIC panels containing standardized two‑fold dilution ranges: imipenem 1–8 µg/mL, meropenem 0.5–8 µg/mL, and ertapenem 0.5–1 µg/mL, as specified in the manufacturer’s panel documentation.” All Enterobacterial isolates were classified into either CRE or carbapenem-susceptible Enterobacteriaceae (CSE) as per the antibiogram. Strains were considered resistant to imipenem, meropenem, or ertapenem if the MIC value was ≥ 8, ≥ 8, and ≥ 1 mg/L, respectively, according to the EUCAST^33^. All CRE strains were confirmed using the E test (Biomerieux, France).

### Molecular characterization of the isolated CRE strains

All phenotypically identified CRE isolates were classified into either carbapenemase-producing Enterobacteriaceae (CPE) or Non-carbapenemase-Producing Enterobacteriaceae according to the presence or absence of carbapenemase genes. The Carba-R assay, using the GeneXpert® system (Cepheid, Sunnyvale, CA, USA), was performed following published protocols for using the Xpert Carba-R assay on cultured isolates^34^.

Briefly, a 40 μL aliquot was directly mixed and vortexed with the provided sample reagent buffer. Then, 1.7 mL was transferred to an Xpert cartridge, which was detected on the GeneXpert platform using* K. pneumoniae* ATCC BAA-1705 (*bla*_KPC_ positive) and *E. coli* ATCC 25,922 (carbapenem-susceptible strain) as positive and negative controls, respectively^35^. This multiplex real-time PCR assay detects the five carbapenemase genes: *bla*_VIM_, *bla*_OXA-48_, *bla*_KPC_, *bla*_NDM_, and *bla*_IMP_. All CPE isolates were classified as A, B, and D classes according to Smith and Kendall^36^.

### Statistical analysis

Data were organized prior to analysis using Microsoft Excel (Microsoft Corp., USA). Descriptive statistics were presented as counts (N) and percentages (%) for categorical variables. Chi square, Monte Carlo, and Fisher exact tests were used for comparative analyses of the AST among CPE and CNE strains.

## Results

A total 814 isolates were identified, classified as non-Enterobacteriaceae (388 isolates (47.7%)), while 426 (52.3%) isolates were identified as Enterobacteriaceae; among them 53 isolates were phenotypically identified as CRE according to the AST breakpoints. All were tested for the presence of the *bla*_NDM_, *bla*_OXA-48_, *bla*_VIM_, *bla*_IMP_, and/ or *bla*_KPC_ genes by the Carba-R assay using the GeneXpert system. A number of 14 strains tested positive for at least one of the five tested genes and were classified as Carbapenemase-positive Enterobacteriaceae (CPE), and 39 strains tested negative for any of the tested genes and were described as carbapenemase-negative Enterobacteriaceae (CNE) (Table 1).

**Table 1:** Distribution of CRE strains across Enterobacteriaceae isolates.

Microbial species	Total isolates (%)*	CRE		
		Total (%)**	CPE (%)	CNE (%)
*Escherichia coli*	243 (57%)	10 (4.12%)	4 (40.0%)	6 (60.0%)
*Klebsialla pneumoniae*	155 (36%)	30 (19.35%)	6 (20.0%)	24 (80.0%)
*Enterobacter cloacae*	10 (2%)	10 (100.00%)	3 (30.0%)	7 (70.0%)
*Providencia alcalifaciens*	3 (1%)	1 (33.33%)	0 (0.0%)	1(100.0%)
*Serratia marcescens*	15 (4%)	2 (13.33%)	1 (50.0%)	1 (50.0%)
Total	426 (100%)	53	14	39

CRE: Carbapenemase-resistant Enterobacteriaceae.

CPE: Carbapenemase-positive Enterobacteriaceae.

CNE: Carbapenemase-negative Enterobacteriaceae.

*Calculated out of 426 isolates.

**Calculated out of the differential species number.

**Fig. 1:**
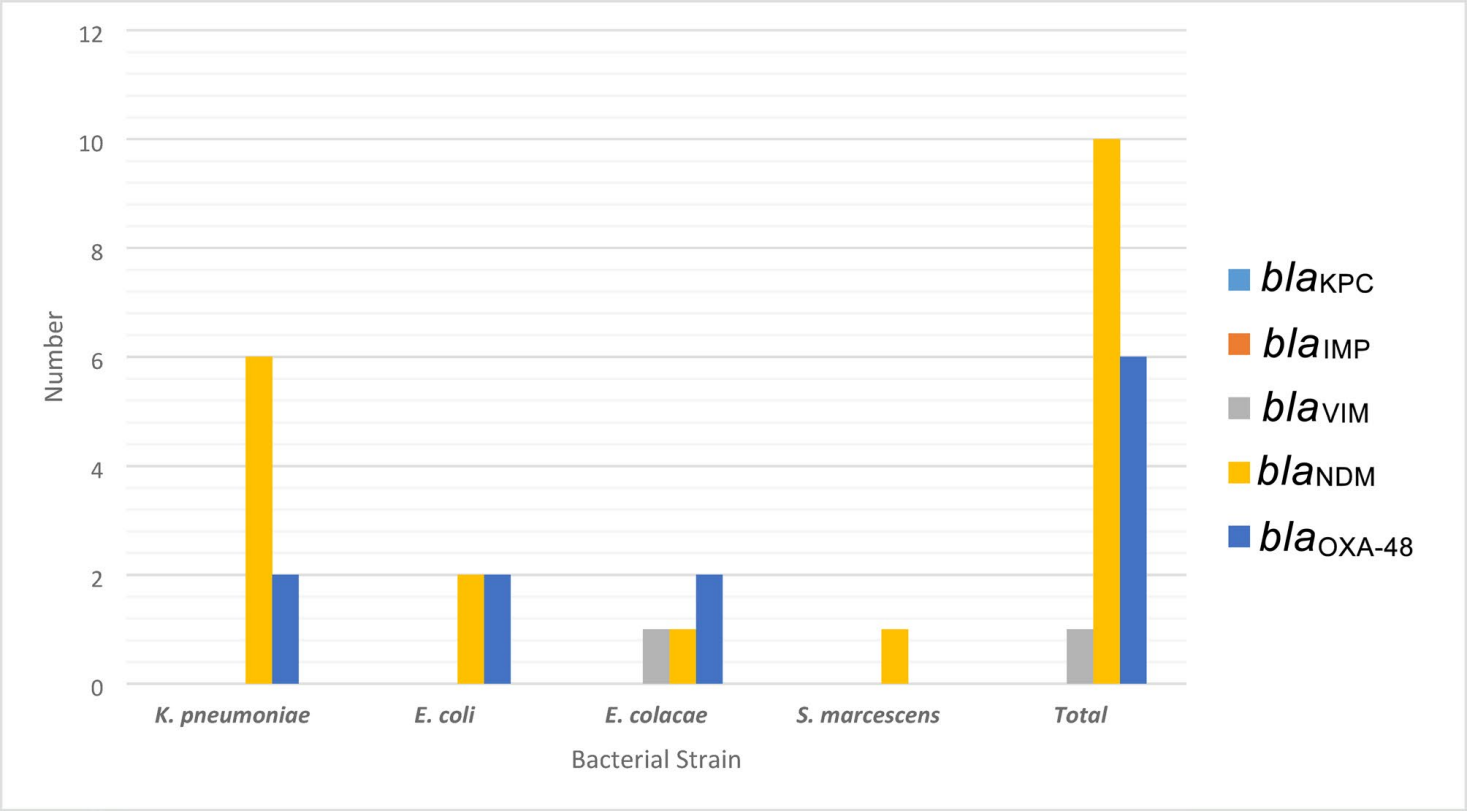
Frequency of carbapenemase genes among carbapenemase-positive enterobacterial (CPE) strains.

*Klebsiella pneumoniae* was the most frequent organism (42.9%), followed by *Escherichia coli* (28.6%), *Enterobacter cloacae* (21.4%), and *Serratia marcescens* (7.1%). Molecular analysis revealed a high prevalence of the *bla*_NDM_ gene (71.4%), with *bla*_OXA-48_ detected in 42.9% of the isolates. *bla*_VIM_ was identified in one case (7.1%), while no isolates harbored *bla*_KPC_ or *bla*_IMP_ genes (Fig. 1).

### Risk factors and epidemiological characteristics of carbapenemase-positive Enterobacteriaceae (CPE)

The age range for patients included in this study was (1–70 years), with median ages of 53 years. Malignancy (28.6%), diabetes mellitus (7.1%), and gastrointestinal neoplasms (7.1%) were found the most common comorbidities among patients. Over one-third (35.7%) had multiple comorbidities. More than half of the patients (57.1%) were admitted to the ICU, while others were distributed across surgical (21.4%), medical (21.4%), and pediatric wards (14.3%). Prior antibiotic exposure was documented in 50% of cases, most frequently involving cephalosporins and Ampicillin–sulbactam (21.4% each), metronidazole and vancomycin (7.1% each). Additionally, 50% of patients were catheterized. The most common sample sources were blood (28.6%), urine (21.4%), wound swabs (21.4%), tracheal aspirates (14.3%), and stool (7.1%). At the time of isolation, clinical diagnoses included sepsis (35.7%), gastrointestinal or respiratory symptoms (28.6%), COVID-19–related illness (14.3%), post-surgical infections (14.3%), and inflammatory disorders (7.1%). Clinical and epidemiological characteristics are summarized in Table 2.

**Table 2:** Epidemiological characteristics of carbapenemase-positive Enterobacteriaceae (CPE) isolates (n = 14).

Variable	n (%)
Age, median (range)	53.0 (1–70)
Female gender	9 (64.3)
Comorbidities	
Malignancy (e.g., lymphoma, colon cancer)	4 (28.6%)
Diabetes mellitus	1 (7.1%)
GI neoplasm	1 (7.1%)
Multiple comorbidities (≥ 2)	5 (35.7%)
No comorbidities	3 (21.4%)
Hospitalization history	
ICU admission	8 (57.1%)
Surgical ward	3 (21.4%)
Medical ward	3 (21.4%)
Pediatrics ward	2 (14.3%)
No prior admission	1 (7.1%)
Prior antibiotic exposure	
Any prior antibiotics	7 (50%)
Cephalosporins (ceftriaxone, cefotaxime)	3 (21.4%)
Ampicillin–sulbactam	2 (14.2%)
Metronidazole	1 (7.1%)
Vancomycin	1 (7.1%)
No prior antibiotics	7 (50%)
Sample source	
Blood	4 (28.6%)
Urine	3 (21.4%)
Wound	3 (21.4%)
Tracheal aspirate	2 (14.3%)
Stool	2 (14.3%)
Invasive devices	
Presence of catheter (urinary or central)	7 (50.0%)
Clinical diagnosis at time of isolation	
Sepsis	5 (35.7%)
GI or respiratory symptoms	4 (28.6%)
COVID-19	2 (14.3%)
Post-surgical infection	2 (14.3%)
Inflammatory disorder	1 (7.1%)

### Distribution of carbapenemase–negative Enterobacteriaceae (CNE)

For the CNE, the identified species were as follows: *K. pneumoniae* (24), *E. coli* (6), *Enterobacter cloacae* (6), *Enterobacter aerogenes* (1), *Providencia alcalifaciens* (1), and *Serratia marcescens* (1), representing 62%, 15%, 15%, 3%, 3%, and 3%, respectively. Among them, 5 isolates (15%), *K. pneumoniae* (4), and *E. coli (1)* tested positive for the ESBL (Table 1).

### Comparison of the AST among carbapenemase-positive Enterobacteriaceae (CPE) and carbapenemase-negative producing (CNE) strains

**Fig. 2:**
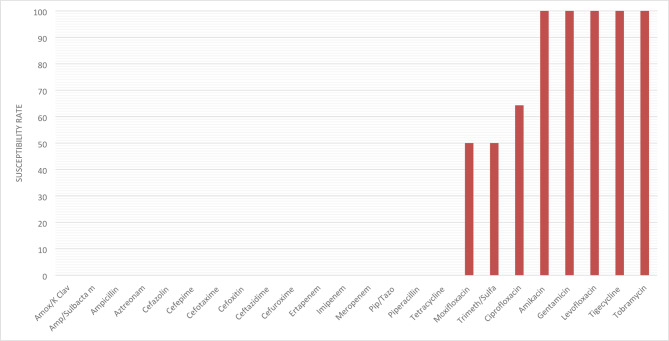
Susceptibility rate of CPE-positive CRE strains to different antibiotics.

High resistance rates were observed among CPE strains. All β-lactams; including penicillins, β-lactam/β-lactamase inhibitor combinations, cephalosporins, and carbapenems; showed no activity against the tested strains. In contrast, fluoroquinolones demonstrated high activity rates (50%, 64%, and 100% for moxifloxacin, ciprofloxacin, and levofloxacin, respectively), while both aminoglycosides and tigecycline exhibited 100% susceptibility” (Fig. 2).

The AST results for CNE strains showed high resistance rates to the commonly used antibiotics, for the penicillin class. On the other hand, they showed low to moderate sensitivity to beta-lactam/beta-lactamase inhibitors: amoxicillin/clavulanate (5%), ampicillin/sulbactam (13%), and piperacillin/tazobactam (18%). In addition, the isolates showed moderate sensitivity to fluoroquinolones, with levofloxacin being the most effective (33%).

**Fig. 3:**
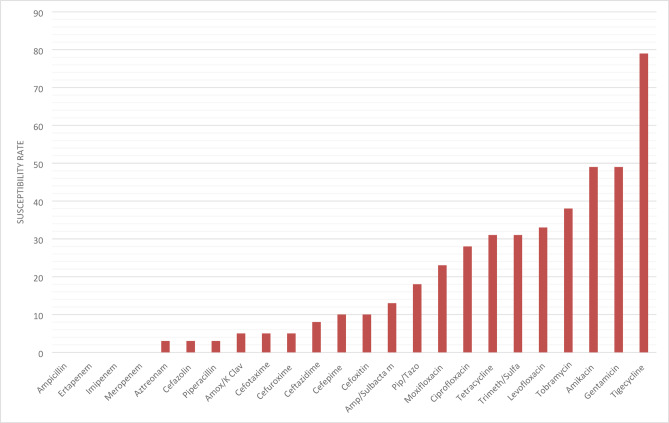
Susceptibility rate of CNE strains to different antibiotics.

Aminoglycosides demonstrated moderate activity against the isolates, with both amikacin and gentamicin showing 49% effectiveness. Among tetracyclines, overall susceptibility to tetracycline was 31%, whereas tigecycline exhibited the highest activity of all tested agents, with a susceptibility rate of 79% (Fig. 3). Statistical comparison revealed significant differences in antimicrobial susceptibility patterns between the groups. Aminoglycoside resistance was detected in 54.7% of CNE isolates, whereas all CPE isolates demonstrated complete (100%) susceptibility to aminoglycosides (*p* < 0.001).

Similarly, a marked variation was noted in β-lactam/β-lactamase inhibitor combinations, including amoxicillin–clavulanate and ampicillin–sulbactam, where all CPE isolates were resistant, but CNE showed susceptibility up to 18% (*p* < 0.001). On the other hand, the penicillin group (ampicillin/piperacillin), resistance reached 98.7% in carbapenemase-producing isolates and 100% among non-carbapenemase-producing isolates, with no significant difference (*p* = 1.0). Similarly, carbapenem agents (aztreonam, ertapenem, imipenem, and meropenem) showed near-complete resistance across both groups (99.4% vs. 100%, *p* = 1.0).

However, cephalosporin class antibiotics (cefazolin, cefepime, and cefotaxime) demonstrated a statistically significant difference, with resistance detected in 93.2% of Group 1 isolates versus 100% in Group 2 (*p* = 0.047). For quinolone antibiotics (ciprofloxacin, levofloxacin, and moxifloxacin), a marked variation was observed between the two groups: 71.8% resistance in Group 1 compared to 28.6% in Group 2 (*p* < 0.001), indicating better quinolone susceptibility among non-carbapenemase-producing isolates. Regarding tetracycline and tigecycline, resistance patterns were similar in both groups (44.9% vs. 50.0%, *p* = 0.066), showing no statistically significant difference.

Finally, no significant statistical difference found between CPE and CNE in their resistance to trimethoprim-sulfamethoxazole (Tables 3 and 4).

**Table 3:** Comparison of antimicrobial susceptibility for each individual antibiotic between carbapenemase-positive (CPE) (n = 14) and carbapenemase–negative Enterobacteriaceae (CNE) strains (n = 39).

		CNE	CPE	*χ* ^2^	*p*
		(n = 39)	(n = 14)		
		No. (%)	No. (%)		
Amikacin	R	20 (51.3%)	0 (0.0%)	14.672*	^MC^*p* = 0.001*
	S	17 (43.6%)	14 (100.0%)		
	I	2 (5.1%)	0 (0.0%)		
Amox/K Clav	R	37 (94.9%)	14 (100.0%)	0.879	^MC^*p* = 1
	S	1 (2.6%)	0 (0.0%)		
	I	1 (2.6%)	0 (0.0%)		
Amp/Sulbacta	R	34 (87.2%)	14 (100.0%)	1.12	^MC^*p* = 0.757
	S	2 (5.1%)	0 (0.0%)		
	I	3 (7.7%)	0 (0.0%)		
Ampicillin	R	39 (100.0%)	14 (100.0%)	–	–
	S	0 (0.0%)	0 (0.0%)		
	I	0 (0.0%)	0 (0.0%)		
Aztreonam	R	38 (97.4%)	14 (100.0%)	0.366	^FE^*p* = 1
	S	1 (2.6%)	0 (0.0%)		
	I	0 (0.0%)	0 (0.0%)		
Cefazolin	R	38 (97.4%)	14 (100.0%)	0.366	^FE^*p* = 1
	S	1 (2.6%)	0 (0.0%)		
	I	0 (0.0%)	0 (0.0%)		
Cefepime	R	35 (89.7%)	14 (100.0%)	1.12	^MC^*p* = 0.673
	S	3 (7.7%)	0 (0.0%)		
	I	1 (2.6%)	0 (0.0%)		
Cefotaxime	R	37 (94.9%)	14 (100.0%)	0.746	^FE^*p* = 1
	S	2 (5.1%)	0 (0.0%)		
	I	0 (0.0%)	0 (0.0%)		
Cefoxitin	R	35 (89.7%)	14 (100.0%)	0.832	^MC^*p* = 1
	S	2 (5.1%)	0 (0.0%)		
	I	2 (5.1%)	0 (0.0%)		
Ceftazidime	R	36 (92.3%)	14 (100.0%)	1.142	^FE^*p* = 0.557
	S	3 (7.7%)	0 (0.0%)		
	I	0 (0.0%)	0 (0.0%)		
Cefuroxime	R	37 (94.9%)	14 (100.0%)	0.879	^MC^*p* = 1
	S	1 (2.6%)	0 (0.0%)		
	I	1 (2.6%)	0 (0.0%)		
Ciprofloxacin	R	28 (71.8%)	5 (35.7%)	7.169*	^MC^*p* = 0.018*
	S	9 (23.1%)	9 (64.3%)		
	I	2 (5.1%)	0 (0.0%)		
Ertapenem	R	39 (100.0%)	14 (100.0%)	–	–
	S	0 (0.0%)	0 (0.0%)		
	I	0 (0.0%)	0 (0.0%)		
Gentamicin	R	20 (51.3%)	0 (0.0%)	15.501*	^MC^*p* = < 0.001*
	S	16 (41.0%)	14 (100.0%)		
	I	3 (7.7%)	0 (0.0%)		
Imipenem	R	39 (100.0%)	14 (100.0%)	–	–
	S	0 (0.0%)	0 (0.0%)		
	I	0 (0.0%)	0 (0.0%)		
Levofloxacin	R	26 (66.7%)	0 (0.0%)	22.796*	^MC^*p* = < 0.001*
	S	11 (28.2%)	14 (100.0%)		
	I	2 (5.1%)	0 (0.0%)		
Meropenem	R	39 (100.0%)	14 (100.0%)	–	–
	S	0 (0.0%)	0 (0.0%)		
	I	0 (0.0%)	0 (0.0%)		
Moxifloxacin	R	30 (76.9%)	7 (50.0%)	6.122*	^MC^*p* = 0.047*
	S	6 (15.4%)	7 (50.0%)		
	I	3 (7.7%)	0 (0.0%)		
Pip/Tazo	R	32 (82.1%)	14 (100.0%)	2.56	^MC^*p* = 0.39
	S	6 (15.4%)	0 (0.0%)		
	I	1 (2.6%)	0 (0.0%)		
Piperacillin	R	38 (97.4%)	14 (100.0%)	0.366	^FE^*p* = 1
	S	1 (2.6%)	0 (0.0%)		
	I	0 (0.0%)	0 (0.0%)		
Tetracycline	R	27 (69.2%)	14 (100.0%)	4.747	^MC^*p* = 0.081
	S	5 (12.8%)	0 (0.0%)		
	I	7 (17.9%)	0 (0.0%)		
Tigecycline	R	8 (20.5%)	0 (0.0%)	6.178*	^MC^*p* = 0.037*
	S	25 (64.1%)	14 (100.0%)		
	I	6 (15.4%)	0 (0.0%)		
Tobramycin	R	24 (61.5%)	0 (0.0%)	28.991*	^FE^*p* = < 0.001*
	S	7 (17.9%)	14 (100.0%)		
	I	8 (20.5%)	0 (0.0%)		
Trimeth/Sulfa	R	27 (69.2%)	7 (50.0%)	1.657	^FE^*p* = 0.198
	S	12 (30.8%)	7 (50.0%)		
	I	0 (0.0%)	0 (0.0%)		

**Table 4:** Comparison of antimicrobial susceptibility among the different antibiotic classes between carbapenemase-positive Enterobacteriaceae (CPE) (n = 14) and carbapenemase-negative Enterobacteriaceae (CNE) isolates (n = 39)).

Antibiotic classes		CNE	CPE	*χ* ^2^	*p*
		No. (%)	No. (%)		
Aminoglycosides		(n = 117)	(n = 42)		^MC^*p* = < 0.001*
	R	64 (54.7%)	0 (0.0%)	53.597*	
	S	40 (34.2%)	42 (100.0%)		
	I	13 (11.1%)	0 (0.0%)		
Amox/K Clav Amp/Sulbacta		(n = 117)	(n = 42)		^MC^*p* = < 0.001*
	R	103 (88.0%)	0 (0.0%)	129.147*	
	S	9 (7.7%)	42 (100.0%)		
	I	5 (4.3%)	0 (0.0%)		
Ampicillin + Piperacillin		(n = 78)	(n = 28)		^FE^*p* = 1
	R	77 (98.7%)	28 (100.0%)	0.362	
	S	1 (1.3%)	0 (0.0%)		
	I	0 (0.0%)	0 (0.0%)		
(Carbapenem classes)		(n = 156)	(n = 56)		^FE^*p* = 1
	R	156 (100.0%)	56 (100.0%)	0.361	
	S	0 (0.0%)	0 (0.0%)		
	I	0 (0.0%)	0 (0.0%)		
Cephalosporin		(n = 234)	(n = 84)		^MC^*p* = 0.047*
	R	218 (93.2%)	84 (100.0%)	5.878*	
	S	12 (5.1%)	0 (0.0%)		
	I	4 (1.7%)	0 (0.0%)		
		(n = 117)	(n = 42)		^MC^*p* = < 0.001*
Quinolones	R	84 (71.8%)	12 (28.6%)	33.323*	
	S	26 (22.2%)	30 (71.4%)		
	I	7 (6.0%)	0 (0.0%)		
Tetracycline + Tigecycline		(n = 78)	(n = 28)		^MC^*p* = 0.066
	R	35 (44.9%)	14 (50.0%)	5.445	
	S	30 (38.5%)	14 (50.0%)		
	I	13 (16.7%)	0 (0.0%)		
Trimeth/Sulfa		(n = 39)	(n = 14)		^MC^*p* = 0.198
	R	27 (69.2%)	7 (50.0%)	1.657	
	S	12 (30.8%)	7 (50.0%)		
	I	0 (0.0%)	0 (0.0%)		

*χ*^2^: Chi square test. MC: Monte Carlo test.

FE: Fisher exact test. *: Statistically significant at *p* ≤ 0.05.

*p*: value for comparing between the two studied groups.

## Discussion

Understanding the regional resistance patterns of carbapenems and the underlying molecular mechanisms is crucial for the design of efficient strategies for antimicrobial drug stewardship. In our study, we conducted a comprehensive analysis of enterobacterial isolates to identify the epidemiological and molecular characteristics of CRE isolates in Jazan region, Saudi Arabia.

### Prevalence and distribution of carbapenemase genes

Our analysis revealed that the prevalence of CRE was 12.4% (53/426) for Enterobacterial isolates, with only 26.4% (14/53) proven to be CPE strains, and the remaining 73.6% were classified as CNE. In a study conducted in Texas from 2011 to 2019, investigators reported the predominance of CNE (59%) over CPE (41%) out of 99 CRE isolates^37^. Higher rate (84.7%) was observed for CPE in a multicenter study by Al Abdely et al. with 90% being *K. pneumoniae* isolates^20^. Higher molecular detection was also observed by Al Raddadi et al. for CPE infections in their study group for the Saudi cohort^21^. A global pooled prevalence rate of 43.06% was reported during 2013 to 2023 across Africa, Asia, Europe and South America^16,38^.

Alhaji et al. reported overall CRE prevalence of 18.58% across Africa, distributed among human, animal, and environment. Highlighted the widespread but under-documented burden of CRE across Africa^17,38^. Our findings suggest that non-carbapenemase mechanisms account for the majority of carbapenem resistance in the Jazan region. Porin mutations, efflux pump, and other intrinsic mechanisms may play a more prominent role than carbapenemase production compared to reports from other regions of Saudi Arabia^25,26,39^, in addition to reports from Africa that highlighted the contribution of non-carbapenemase mechanisms (ESBL/AmpC + porin loss in CNE isolates^17,18^, especially 15% of our CNE strains were found to be ESBL. The inter-regional variability could be attributed to the selective pressure due to antibiotic usage and implementation of different antibiotic stewardship programs^3^. Additionally, the inter-regional variability in patient population, including travel and healthcare-seeking behaviours, affecting the entry and spread of resistance gene carriers^8^. Moreover, infection control practices and hospital epidemiology could provide different ecological niches to non-carbapenemase resistance mechanisms^18,40^.

### Molecular epidemiology of the CPE isolates

Out of the 14 CPE strains, *bla*_NDM_ was found to be the most prevalent carbapenemase gene (10 isolates, 71.4%), followed by *bla*_OXA-48_ (6 isolates, 42.9%), while only one *bla*_VIM_-positive (7.1%) strain was obtained. However, neither the *bla*_KPC_ nor *bla*_IMP_ was detected. These findings are consistent with multiple reports in Saudi Arabia that documented the predominance of *bla*_OXA-48_ in tested strains^9,22,23,41^. A multicenter study conducted in 16 hospitals in four Gulf countries found that the *bla*_NDM_ and *bla*_OXA-48_ were the common genes behind emergent resistant strains^8^. Another cohort study in Saudi Arabia reported the *bla*_OXA-48_ as the most prevalent gene among CRE implicated in bacteremia, resulting in mortality^21^. A study conducted by Taha et al. found that *Klebsiella* and *E. coli* were the commonly identified CRE genera in Jeddah city over 3 years from 2017 to 2019, moreover, they reported that the *bla*_OXA-48_ and *bla*_NDM_ genes were the common cause of resistance among the isolates^27^. A report from Africa demonstrated that Africa shares a similar global high-risk resistance mechanism with a predominance of *bla*_NDM_, *bla*_OXA-48_ among CRE strains^16,17^. Similarly, the dominance of OXA-48–like enzymes aligns with global trends in the Middle East–North Africa region as reported by Alhaji et al. and Olaitan et al.^18,42^. The predominance of *bla*_NDM_ detected in Jazan is likely influenced by regional epidemiological dynamics; Jazan’s proximity to Yemen, combined with its role as a transit hub for individuals originating from South Asia; where *bla*_NDM_ is highly endemic; may facilitate the cross-border introduction and dissemination of NDM-producing Enterobacteriaceae^5,32^. Particularly, India and Pakistan are recognized hotspots for the *bla*_NDM_-harboring strains, the continuous flow of migrant workers, religious pilgrims, and travellers between these countries and southwestern Saudi Arabia may support the dissemination of such strains within healthcare facilities in Jazan^8^. The absence of *bla*_KPC_ and *bla*_IMP_, together with the low detection of *bla*_VIM_ in our isolates, is consistent with regional epidemiological patterns^20,41,43,44^. Co-harboring of both *bla*_NDM_ and *bla*_OXA-48_ genes detected in *K. pneumoniae* isolates is alarming concern, such multi-gene profiles markedly restrict therapeutic options by conferring multi resistance to nearly all β-lactam agents. Such findings agree with regional reports of multi-carbapenemase plasmid co-carriage combinations among *K. pneumoniae* isolates^15,45^.

### Species distribution patterns and ward-specific variations

The predominance of *K. pneumoniae* among both CPE isolates (42.9%, 6/14) and CNE strains (61.5%, 24/39) is consistent with regional and global distribution patterns previously reported in surveillance datasets^20,21,46^. Similar findings were reported in Africa by Alhaji et al.^18^. The intrinsic characteristics of *K. pneumoniae* might contribute to its dominance; efficient acquisition of resistance plasmids, robust biofilm formation, and strong environmental persistence^5^. Being a frequent gastrointestinal commensal and a significant community reservoir of antibiotic resistance genes, *E. coli* also accounted for a significant percentage of CPE isolates (28.6%, 4/10).

Regarding the ward-specific distribution patterns of *bla*_NDM_- and *bla*_OXA-48_ positive strains, the *bla*_NDM_ was mainly linked to the ICU admission, whereas *bla*_OXA-48_ was most frequent in pediatric wards. Such patterns may be attributed to different selective pressures by antibiotics, as well as patient population variations^47^. ICUs usually have more frequently utilized antimicrobial agents and more critically ill, invasive-device-borne patients, hence predisposing isolation and colonization by *bla*_NDM_-producers^6,40^. Isolation of *bla*_OXA-48_ in pediatric words might be suggestive of clonal spread or contamination^6,48^.

### Antimicrobial susceptibility

The susceptibility profile revealed highly resistant phenotypes with major therapeutic implications. In align with other report^3,49^, all CRE isolates were uniformly resistant to penicillins and carbapenems. β‑lactam/β‑lactamase inhibitor combinations, and the monobactam aztreonam showed minimal activity, which were associated with frequent co‑expression of ESBL. The poor activity of aztreonam most likely due to the hydrolyzing capacity of ESBL in positive strains despite its stability against metallo‑β‑lactamases^9,23^, such a finding raise a concern on the potential of aztreonam–avibactam therapies whose efficacy might be attenuated in ESBL‑rich settings^28,50^. Fluoroquinolone susceptibility remained mild (33%), limiting their empirical utility as a monotherapy^3,40^. Nevertheless, aminoglycosides showed better activity (49% for amikacin and gentamicin), supporting their use in combination regimens despite the toxicity concerns^10^. Tigecycline exhibited an overall susceptibility rate of 79%, aligning with regional reports of up to 90% activity against *K. pneumoniae* CRE^3^. Such activity could be attributable to its unique ribosomal binding mechanism, and reduced vulnerability to efflux‑ and enzyme‑mediated resistance^10,51^.

### Comparative analysis: CPE vs. CNE strains

The findings reveal a significant difference between CPE and CNE in their multidrug resistance patterns. Overall, the CPE strains exhibited significantly higher multidrug resistance, including complete resistance to all β-lactam/β-lactamase inhibitors, that could be attributed to the co-selection of resistance genes on mobile elements^15,37^. CPE strains showed complete susceptibility to aminoglycosides, compared to 49% of CNE strains. However, CPE showed significantly lower resistance rates (28.6%) to quinolones compared to CNE (71.8%; *P* < 0.001), offering potential treatment options. Carbapenem resistance continues to be a major therapeutic problem, as evidenced by common resistance to β-lactams^25,37^.

## Conclusion, strength, limitation, and future work

This study provides insights into the molecular epidemiology of carbapenem-resistant Enterobacteriaceae in Jazan region, addressing an important gap in national surveillance of antimicrobial resistance. Our findings highlight the predominance of non-carbapenemase mechanisms, emphasizing the unique epidemiological characteristics of the area and the consequent implications for targeted stewardship and infection control strategies. The NDM and OXA-48–like enzymes are found to be the principal drivers of carbapenem resistance among CPE. A key strength of this work lies in its broad inclusion of all clinically relevant Enterobacterales (*Klebsiella, E. coli, Citrobacter, Enterobacter…*etc.), unlike previous reports that focused primarily on *Klebsiella spp.* Limitations: the single-center setting and relatively short study period limited the sample size. In addition, non-carbapenemase mechanisms were not molecularly confirmed. Prospectives for future research: Further investigations to explore different underlying resistance pathways beyond carbapenemase production across diverse clinical settings and provinces in Saudi Arabia to better guide national containment efforts.

## Data Availability

The data that support the findings of this study are available from correspoding author (Gharieb S. El-Sayyad), but restrictions apply to the availability of these data, which were used under license for the current study, and so are not publicly available. Data are however available from the authors upon reasonable request and with permission of corresponding author.
